# Structural Discrimination in Physical Education. The “Encounter” Between the (White) Norwegian Teaching Content in Physical Education Lessons and Female Students of Color's Movements and Expressions

**DOI:** 10.3389/fspor.2021.769756

**Published:** 2021-11-15

**Authors:** Erik Aasland, Gunn Engelsrud

**Affiliations:** ^1^Department of Sport Science and Physical Education, University of Agder, Kristiansand, Norway; ^2^Department of Sport, Food and Natural Sciences, Western Norway University of Applied Sciences, Bergen, Norway

**Keywords:** race, class, ethnicity, physical education, critical whiteness, embodiment

## Abstract

**Background:** Throughout society, including in the field of sports and physical education (PE), there are extensive debates on racism and structural racism. Researchers have found that students of color experience racial stereotyping and discrimination in PE. Studies also show that PE teaching practices reflect a masculine culture, emphasizing traditional (Western) competitive male sports and physical fitness practices, while marginalizing female students of color. Simultaneously these students also negotiate, resist, and operate as visible agents within these practices. While previous research has tended toward focusing on the experiences (of discrimination) from the perspective of students of color, numerous scholars today argue for a shift in research focus from the inadequacies of the racialized “others” toward how (white) everyday pedagogical practices privilege some people, and conversely, marginalize and discriminate against students of color. We believe, however, that it is crucial to examine and direct attention toward the students who “encounter” discriminatory practices in PE.

**The Purpose of the Paper is Twofold:** (i) To investigate what kind of movements, knowledge/skills and bodies are valued and privileged, or ignored/discriminated in teaching practices in PE. (ii) To depict the “encounter” between the teaching content in physical education lessons and the female students of color's movements and expressions.

**Research Design and Production of Empirical Material:** Our sample consisted of institutionalized pedagogical practices enacted by four white PE teachers in an upper secondary school located in eastern Oslo, Norway. The empirical material consisted of observations and informal conversations extracted from 42 teaching lessons. Students from a great diversity of backgrounds participated in the lessons during our study. We chose a critical whiteness perspective and critical feminist theory as analytical lenses for the analyses.

**Findings:** Strenuous exercise as a means of becoming physically fit and “healthy” was central during the PE lessons under observation. The teacher's instructions draw on healthism discourses influenced by knowledge from exercise physiology and biomechanics, which produce a normative ideal for the body and movements in PE teaching practice. The movements and expressions of female students of color did not meet the expected norm of intensity, activity and exercises from the health and sport discourses. However, we identified female students of color as competent movers when performing “their own” movements, with which they had more of a cultural connection. These students also expressed vitality, joy and social interaction. Thus, they created new movements and expressions that contrasted with the norm of working out with a high intensity level in PE lessons.

**Conclusion:** Hegemonic “truths” about the body, physical exercise and health make teachers “blind” to the ways that institutionalized pedagogical practices in PE privilege majority ethnic white (male) students. Refusing to adhere to the institutionalized movement/exercise practices enacted by the teachers, the female students of color are competent dancers and express liveliness, spontaneity, and joy. In the future, incorporating these types of expressions to teaching practices can add value to PE.

## Introduction

Since the death of George Floyd in May of 2020, the Black Lives Matter movement has increased awareness of racism throughout society, and recently^1^ there have been extensive debates on structural racism. Even though debates on race and racism are not new, discussions about structural racism^2^ have accelerated and indicate a new awareness of how the laws, rules and politics of a society perpetuate unfair advantages for certain groups of people based on their race or ethnicity. In this paper we will address the issue of structural racism in Norwegian physical education (PE). While there is no direct connection between the case of George Floyd and PE in Norway, we feel that the murder of George Floyd can shed light on the ways that the power to define and value different bodies based on their skin color is so deeply embedded in systems of thought that it often goes unnoticed. Our intention is to search for ways that this type of prejudice is present in the structure of the Norwegian school system. In PE, pupils are expected to move in certain prescribed ways, and this is confirmed in teaching practice. In this article, our aim is not only to illuminate unfair treatment of pupils based on color and race in PE, but to show how certain pupils are defined as “the other” by the structures in which teachers are embedded. This will be an opportunity for us to criticize and problematize the traditional (white) context and content in PE. We build on literature that clearly addresses how students of color^3^ are treated unfairly or overlooked by their PE teachers. However, as Barker (2019), as well as Simon and Azzarito (2019) argue, there are interesting intersections between racial and gender discourses that deserve more attention. Several studies show that a sport-focused and masculine PE curriculum does not serve the interests or the identity of female students of color (Hill and Azzarito, 2012; Benn and Phister, 2013; Thorjussen and Sisjord, 2018). Therefore, we choose to focus on female students of color, who seem to be particularly vulnerable in the PE teaching practices. We think it is crucial to direct our attention toward the pupils who encounter discriminatory practices, and to try to understand how they negotiate the movement culture to which they are subordinated. However, we do not wish to portray these students as mere victims of institutional racism. Instead, our work shows the ways in which female students of color subvert the values of a teaching practice based on competitive sports, endurance, and intensity, by finding their own ways to move and express themselves. Our aim in this article is twofold:

(i) To investigate what kinds of movements and bodies are valued and privileged, or the opposite, in PE teaching practice.
(ii) To depict the “encounter” between the teaching content in physical education lessons and the female female students of color's movements and expressions.

## Previous Research and Knowledge Status

In the field of sports and physical education, researchers have found that Black students and students of color experience racial stereotyping in both PE classes and PE teacher education (PETE) (Azzarito, 2009; Douglas and Halas, 2013; Hylton, 2015; Dowling and Flintoff, 2018; Flintoff, 2018; Barker, 2019; Flintoff and Dowling, 2019). Azzarito (2009), for example, has shown that the PE teachers promote a racialized conception of the ideal female body. The study showed that students identified the ideal female body as white, and that African American women were placed at the bottom of the body gender hierarchy (Azzarito, 2009). Furthermore, Azzarito (2019) argues that in Western globalized societies, fit and healthy bodies represent “valued bodies.” In contrast to valued bodies, Azzarito (2019) suggests that “bodies out of sight” are bodies positioned outside normative ways of thinking about fitness and health. She suggests that the “bodies out of sight” are predominantly people of color whose bodies occupy a marginal position in today's mainstream white landscape of fitness and health.

Coming back to the structural aspects of racism, even though public institutions like schools have a mandate to be democratic and strive for inclusionary practices, research literature suggests that teachers, teacher educators and students are “blind” when confronted with structural racism and discrimination in PE (Douglas and Halas, 2013; Barker, 2019; Flintoff and Dowling, 2019). According to the research done by Douglas and Halas (2013), as well as Flintoff and Dowling (2019), PE teacher educators and students consist largely of white people, and the curriculum is formed by people who have often never experienced racism in their own lives and therefore fail to consciously address racism in their work. Based on this evidence, Douglas and Halas (2013) argue that racism is produced through silence, invisibility, and exclusion, and that predominantly white faculties and student bodies often fail to recognize themselves as racially structured environments. Furthermore, as Flintoff and Dowling (2019) argue, it is *particular* abilities and bodies that are valued and promoted, a phenomenon closely linked to ideas of national identity and notions of “our” shared physical culture that are easily taken for granted. They use an example from a Norwegian teacher educator's narrative about an African student—black, overweight and without any skiing experience—who posed critical questions about why it is mandatory for all students to participate on a day out skiing. The educator's response was that the ski day had been planned since the beginning of the school year. However, (s)he felt a bit unsure about what to say and do in relation to the student. In his/her dialogue with the researchers, the educator stated: “I don't think we should stop having a ski day, just because some students don't like the idea of it and think it's difficult.” In this situation, the educator, who was white and ethnically Norwegian, found it difficult to problematize or think critically about his/her own knowledge, and experience of skiing (although there might be good reasons to have a mandatory ski day, the educator's response seems more like a knee-jerk defense of traditional values than a thoughtful consideration of the student's perspective). The educator chose to normalize the assigned activity, thereby positioning students who lack prior experience as the “problem.” In their analyses, Flintoff and Dowling (2019) position the African student as a “body out of place.” Thorjussen and Sisjord (2020, 58), explain that the idea of “our” shared physical culture “largely reflect(s) Western^4^ discourses around sport, fitness, and health.” This is apparent in Norwegian PE teaching practice, where teacher's choices of activities tend to be a direct reflection of the majority culture. Activities like baseball or cricket, which many students of color have prior experience with and would like to play, are neglected in PE class. Thorjussen and Sisjord (2020) argue that this is a result of the teacher's tendency to orient their pedagogical approach toward activities that they uncritically accept as part of “our” shared knowledge. Consequently, in their study, Thorjussen and Sisjord conclude that non-Western movement cultures are marginalized and excluded in PE.

As the research literature demonstrates, students of color experience structural racism in PE. However, Gullestad (2002) and Flintoff (2018) both suggest that critical scholars have tended to focus too much on the experiences of the racialized “others,” rather than on how standard (white) pedagogical practices develop by reproducing racial stereotypes and discriminatory practices. Shifting research focus away from the inadequacies of the racialized “others,” toward how whiteness discourses work to normalize pedagogical practices that privilege some people over others, can help us understand how structural or institutional discrimination of the “other” unfolds in practice. Dowling and Flintoff (2018), for example, have used a critical whiteness perspective in their analyses of national curricula texts for Physical Education in the UK and Norway. They argue that the learning objectives in the Norwegian PE curriculum are mainly constructed as implicitly ethnic practices, in which activities like skiing and skating are reinforced as normal and valued practices in Norway. The curriculum document also normalizes the learning objects of health and fitness, without taking into consideration perspectives of social class, gender, and ethnicity. Barker (2019) also employs a critical whiteness perspective and argues that PE teachers in Sweden draw extensively on the racial discourse of whiteness, and thus normalize the idea that students of color have a substandard understanding of acceptable behavior, different conceptions of healthy lifestyles (less healthy) and lack motivation (they did not care about grades). However, according to Barker (2019), teachers made sharp distinctions between female and male students from other cultures. They saw girls as “losers” in situations where culturally defined feminine norms were set up against other culturally defined norms that circumscribe participation in PE and sport. In contrast, according to the teachers, boys were “strong enough” to resist the education being provided, they were more likely to overestimate their physical ability, and they saw themselves as superior to girls.

As indicated in Barker's study, PE teachers perceived female students of color as “losers” compared to the boys. Several studies find that PE remains a gendered practice, reflecting a masculine hegemony (Flintoff and Scraton, 2001; Fagrell et al., 2012; van Amsterdam et al., 2012; Scraton, 2018; Aasland et al., 2020). Surveys in Norway show that girls report a less positive attitude toward PE than boys do (Säfvenbom et al., 2015; Moen et al., 2018). Gender differences, which in these studies are used as biological binary categories, are highest among students with little leisure sport experience—a result of the subject content being dominated by traditional sports and physical fitness activities (Säfvenbom et al., 2015; Moen et al., 2018). Because the subject content of PE still consists largely of what are traditionally perceived as masculine activities (Redelius et al., 2009), it encourages students to act like “typical boys” (Aasland et al., 2020). Studies investigating how religion (Walseth, 2015) and ethnicity/race (Thorjussen and Sisjord, 2018) influence students' experiences of being included/excluded in PE, find that gender might have a greater influence than religion and ethnicity.

To sum up, previous research shows that whiteness discourses shape “our” shared physical culture and determine which types of bodies and movement are valued. Consequently, as Barker et al. (2014) point out, an analysis of race and ethnicity in PE lessons would help to address the ways that whiteness and ethnicity discourses foster structural blindness, allowing racism and discrimination to be performed unconsciously in the system of values and privileges that govern movements and actions in PE lessons. However, previous research also indicates differences between colored students' experiences, in terms of how the students choose to negotiate or resist the pedagogical structure of their classes (Hill and Azzarito, 2012; Fitzpatrick, 2013; Barker et al., 2014; Stride, 2014; Walseth, 2015; Thorjussen and Sisjord, 2018). Hill and Azzarito (2012), for example, noted that involvement in PE lessons offered British Asian females few alternatives for producing their own subjective narratives as valued sporting bodies. However, outside of school these girls found their own ways to engage in physical activity, pushing back against notions of Asian women as passive and physically inactive. Yet, as we read and interpret much of the research literature, the racialized “other” are too often seen as “deficient” in relation to a white norm, and thus, these students are portrayed as individuals without the ability or agency to resist structural racism and discrimination. In this regard, we find inspiration in the work of Ahmed (2017). In *Living a Feminist Life*, she uses the concept of “willful girls” (2017, 66) to spotlight girls with a strong subjectivity. Ahmed argues that subjects (willful girls) have the potential to “fight back” and resist discrimination.

## Theoretical Perspective

We chose a critical whiteness perspective and critical feminist theory as analytical lenses in our investigation. Traditionally, race refers to the “other,” not to the people with light colored skin who employ the term. However, according to Gullestad (2002), in order to understand racism, it is not sufficient to study “the other.” To get the “whole picture,” researchers must investigate the majorities who represent the traditional norms within a society and the entire relationship between majorities and minorities. In her analysis, Gullestad (2002) shows how historical and cultural systems of thought make everyday harassment and discrimination of minorities possible. Likewise, feminist scholars of color (Hooks, 1997; Ahmed, 2007) have argued that conceptions of “whiteness” have not generally been seen as historical and social constructions in the same sense as other racial denominations, and that this invisibility serves certain social interests. Frankenberg (1993), a white British sociologist, used this perspective when she interviewed white American women about their life stories. In their stories, she found implied constructions of “whiteness” within discourses of race. Racialization affects both white and black people, though in different ways. According to Frankenberg (1993), the women's “whiteness” was an empty category, the unmarked normative center that *singles out the others*, meaning those who are constructed as different (from the whites). In other words, hegemonic beliefs and practices about race tend to position whites as “normal” and racially “unmarked,” while “others” are defined and seen as “deficit” and “named” (Flintoff, 2018, 209). Moreover, according to Flintoff (2018), whiteness does more than just marginalize the racialized “other.” Whiteness also serves to create a set of social advantages for white people. However, these structural advantages are difficult for whites (and others) to see or acknowledge. Many white people—including PE teachers—are “blind” to normalized pedagogical practices that privilege some (white, middle-class) students, while relegating “others” (female students of color) to being “problems” or “bodies out of place,” marginalized and discriminated against in the PE context.

Adopting a critical whiteness perspective entails acknowledging and challenging the invisibility of whiteness. Gabriel (1998 in Flintoff, 2018) argues that this invisibility is maintained using different discursive tools. The discursive technique *naturalism*, for example, concerns itself with how race is defined in relation to “others,” while white bodies and perspectives are considered “natural” and society's standards of beauty, fitness and other indicators of value are based on them. The discursive technique of whiteness as *universalization*, engenders the view that white experience and knowledge represent the experiences of everyone, thereby denying the presence and impact of racism on the lives of the racialized others. With this understanding, the critical whiteness perspective can give us an opportunity to uncover how habitual power privileges some students' bodies and movements, and inversely, marginalizes other pupils' movements and expressions.

As our review demonstrates, several studies show that girls in PE are marginalized. Feminist theory can help us understand whether and how gender inequalities and/or differences impact the experiences of students, especially girls, in school PE classes (Scraton, 2018). In her paper, “Feminism(s) and PE: 25 years of Shaping Up to Womanhood,” Sheila Scraton (2018) writes that there have been different “waves” of feminism, but “new” feminists are now looking for “a ‘middle ground' approach that recognizes persistent material inequalities as well as the nuances and fluidity of individual lives” (647). Despite many female students of color's experience of discriminatory practices in school and in their daily lives, many of them are still agents in their own lives. Azzarito (2010), for example, identifies the construction of “new” types of femininity among girls diverge from traditional notions of the docile female body. Recent studies also reveal that PE is sometimes an arena where girls can challenge gender stereotypes (Walseth, 2015; Aasland et al., 2020). Girls who resist and challenge gender stereotypes and inequalities are examples of people whom Ahmed (2017) would have called “willful subjects.” Ahmed argues that when girls (or “others”) exercise their own will, they are deemed willful. Willfulness is closely related to stubbornness, obstinacy, and disobedience. Labeling people as willful is a way of reproaching them for having too much will, or too much subjectivity. Ahmed finds a lot of stories of willful girls in literature, and, as she notes, such histories are still being told. The idea of the willful child functions to justify violence against and the elimination of willfulness in these people. Nevertheless, as Ahmed (2017) suggests, willfulness represents persistence in the face of oppression, and continued persistence and disobedience mean that hope lives on.

## The Norwegian Pe Curriculum

According to the national curriculum for PE^5^, the purpose of PE in Norwegian schools is to inspire physical activity in all aspects of life and a lifelong enjoyment of being physically active, and “… the subject aims to provide the students with physical challenges and the courage to defy their own limits, in both spontaneous and organized activities. The training is to preserve both traditional and alternative movement activities in the subject, and to stimulate experimental and creative development” (Utdanningsdirektoratet, 2015).

The PE curriculum in upper secondary level covers a broad content area, but the primary focus is on sports activities, outdoor activities, and exercise (Utdanningsdirektoratet, 2015). Within these content areas there are competence goals the PE teacher is obliged to teach students and use in their assessment. The competence goals describe the expected learning outcomes that primarily center around each student's competence in *practicin*g and *carrying out* movements and exercises—as well as their comprehension of and reflections on targeted areas related to physical activity, the body, and health. Competence goals are not formulated in a way that indicates that the student should develop their physical fitness (physical strength, endurance, etc.). However, there are few specifications regarding the content and methods in the PE curriculum, so selection of the sports and activities (content) that are taught, is to a large extent the responsibility of the individual PE teacher. Prior research shows that health and sports discourses are indicative of the content and methods in Norwegian PE. A national survey, for example, showed that traditional (ball) sports and physical exercise activities dominate the teaching content in PE, while outdoor activities (Norwegian friluftsliv), lifestyle sports and dance have a minor influence in teaching practice (Moen et al., 2018).

## Research Setting and Methods

To investigate how (white) everyday pedagogical practices privilege some students, and conversely, marginalize and discriminate “others,” we use material^6^ from one upper secondary school located in eastern Oslo. Oslo is developing into an increasingly multiethnic city; 32% of the city's population are immigrants; the percentage is even higher on the eastern side of the city. Our sample consists of institutionalized pedagogical practices (42 PE lessons) enacted by four white, ethnic Norwegians PE teachers, whom we have assigned the pseudonyms Jane, Roger, Henrik, and Thomas. During our research, Jane proved to be the most pedagogically and socially active of the teachers and we found ourselves in several spontaneous conversations with her. Consequently, Jane is more prominent than the other teachers in our findings. The age of the teachers varied between 30 and 55 years at the time when the Ph.D. project took place. Each of the teachers had at least 180 ECTS credits in sports and/or PE. We followed these four teachers in different PE classes. The PE classes are gender mixed and consisted of 15–20 students. In many of the classes more than 50% of the students were students of color. We provided the teachers and students with oral and written information about the project prior to obtaining their consent for participation. The Norwegian Center for Research Data approved the project.

Usually, three classes had PE at the same time with their respective teachers. However, the three classes often mixed with each other during lessons. One frequent teaching structure consisted of a warm-up sequence (mostly jogging in circles in the sports hall) and workout exercises in the sports hall (for example Tabata), that were the same for all the students. Next, students could choose to play football, floor hockey or basketball in different areas in the sport hall, to exercise on the machines, or to box or dance in the mirror-room. According to the teachers, many students choose to play ball sports and from the point of view of the teachers, these sports provide high intensity training. These are the general frames of the structure set up by the teachers, but within this structure there are numerous situations that go unnoticed, activities that are marginalized in the PE culture (teaching practice) but continue to occur, nevertheless.

### Production of Empirical Material and Selection of Situations

The material consists of notes taken while observing 42 PE classes. Conversations with the teachers occurred during observation of nearly every class—usually both before and after a lesson, sometimes during them as well. Spontaneous conversations between the first author and various students also occurred during many of the lessons. The first author took a relatively open approach to fieldwork, paying attention to what students and teachers said and did during PE lessons (Alvesson and Kärreman, 2005; Fangen, 2010). Despite this freeform approach, the observations were informed by the discourse perspective and critical PE literature. Questions that guided first author while conducting the fieldwork were: What content and activities do the teachers introduce? In addition, what activities do the teacher emphasize in teaching practice? What knowledge do the teachers present as valuable?

Previously in this article we have discussed the invisibility of whiteness. We recognize that, as white researchers, our work occupies an occasionally uncomfortably tight space between challenging white privilege and awareness of our own epistemological frame of whiteness. This means that, like the other PE researchers we refer to, we must take into consideration the majority–minority power structures and the global history of colonial and neo-colonial relations in order to place our perspective in its proper context (Gullestad, 2004). For the following analyses we have singled out situations from institutionalized pedagogical practices in PE, in which the white ethnic Norwegian teachers and some of the female students of color struggle to communicate about the value of different types of movement. We based our choice of situations to analyze on narratives and notes from the observations. The analysis of the material is processed and mediated through our emotional responses, as well as our theoretical lenses, which developed as we became aware of these female students' vitality, the way they and their actions were overlooked by their teachers, and their apparent agency and willfulness in the face of a PE class that did not seem to value their contributions.

## Findings

We have organized our findings under four headings: (i) Strenuous exercise as a means of becoming physically fit and “healthy,” (ii) female students of color's “meeting” with prescribed exercises, (iii) when singing and dancing are out of place: a failure to understand the “rules,” and (iv) dance instructions and preferences.

### Strenuous Exercise as a Means of Becoming Physically Fit and “Healthy”

As in many Norwegian PE classes, the teachers in our study often chose strenuous exercise as the content in the lessons. Performing assigned exercises was considered mandatory, and to receive a grade, the students had to accept and perform the activities. The exercises consisted generally of Tabata-activity, running on stairs, strength exercises in the sports hall and use of the exercise machines. Generally, the pupils enter the spinning classroom and start rowing, running, or cycling while the teachers give their instructions, such as this information given by Roger at the start of a class: “We will stay here and continue to either cycle, run or row for at least 15 min. Afterwards you will get some assignments from me, which you are to complete either here (in the spinning classroom) or in the strength training area. While the pupils, cycle, run or row, Roger is busy writing on the blackboard that the students must complete the following exercises: “The Plank” for 5 min, 50 repetitions of pop-ups, 100 repetitions of sit-ups, 70 repetitions of push-ups, 90° supported against a wall for 4 min, and 25 repetitions of diagonal push-ups. He finished writing, looked at the pupils and told them that “today it's important to try to push yourself.” Then he approached two of the female students of color on the rowing machines and told with a serious tone: “Girls. It is smart of you to make a serious effort.” He walked quickly away and left the girls to themselves. One of the girls said quietly to the other one: “This is really boring.”

In this session, Roger assigns his students strenuous exercises to be completed. In an abundance of research literature on PE (Aasland et al., 2017; Larsson and Nyberg, 2017; Moen et al., 2018), using strenuous exercises as favored content has been both criticized and problematized. Roger's lesson plan appears to disregard this research, reaffirming the normalization of strenuous exercises. Informing the females that being smart means pushing yourself is an infantilizing form of communication. The teacher did not wait to hear a response from the girls or ask for their opinion on the assigned exercises. To be “smart,” the girls completed the obligatory exercises, even though they found them to be boring.

After observing the classes over a period of time, it became apparent that they were organized around strength training, weightlifting and exercises intended to build strength in various muscle groups. We observed repeatedly that the female students of color appeared to be unfamiliar with this type of exercise and displayed somewhat “unusual” running movement patterns when performing the pre-defined physical fitness exercises that the teachers had instructed them in. The exercises were designed to orient the body “into the exercise” and move “arms and legs in “correct directions.” Often the girls claimed that the exercises were “too hard.” Henrik and Roger's typical response was “that's good. It's supposed to be hard. Imagine if you were doing this three times a week. Then you would get in good shape.” “Come on! Don't give up! Well done!”

These experiences from our observation notes illustrate that vigorous and strenuous exercises dominate the teaching lessons. Strenuous exercise (strength and endurance) is normalized within teaching practices and embedded in influential discourses of exercise physiology and physical fitness, based on assumptions of health and fitness as universalized knowledge (Tinning, 2010). Students who have internalized this understanding of exercise and health treat these practices as “normal” and they are habituated to respond to the practice as appropriate in PE classes. From our perspective, they have not been introduced to other ways of practicing PE, this lack of alternatives contributes to their discipline and willingness to perform these exercises. In contrast, the students who dislike these exercises struggle to find them relevant or fail to perform them in accordance with the teachers' expectations regarding level of intensity, are deemed unwilling and uncooperative by the teachers. Azzarito et al. (2017) and Azzarito (2019), contend that the dominant narratives of “fit” and “healthy” bodies that are given value in Western globalized society privilege white (upper middle class) people. As a result, according to Azzarito (2019), within these narratives that describe a fit and healthy body, many young people of color are situated as “bodies out of sight.”

### Female Students of Color's “Meeting” With Prescribed Exercises

The following situation demonstrates how female students of color's movements and expressions are seen as “out of place.” The female teacher Jane instructs the students to rotate through different strength-training stations: squat jumps, abdominal exercises, back muscles, and step work. Each station has an assigned number of repetitions per set, a typical characteristic of this type of exercise. I (the second author) followed three females of color into the room where they were supposed to do squat jumps. Jane told the girls to fetch some mats from the equipment room, put them on the floor and do five sets of ten repetitions.

The girls did not start the exercises right away, instead they went to get some floor mats from the equipment room. The girls rambled into the equipment room, bumping into each other, and laughing. The mats were big, yellow, and plastic, with a slightly slippery surface. They carried one out between the three of them and tossed it onto the ground, almost being pulled down themselves in the process and laughing all the while. The mats did seem to resist being moved. Dealing with the mats delayed and diverted the girls from doing their squats. They hauled another mat out of the equipment room and heaved dragging the mats around, trying to get them to lay straight, edge on edge. The mats were difficult, unwilling—in a way, reminiscent of the girls' bodily unwillingness to perform the assigned exercises. Jane came into the room and told them “it's time to get started, no more messing around! Start your exercises!” The mats still didn't lay straight, and the girls tried to turn them to face the other direction in the room, giggling as they struggled. Finally, they began their squat jumps. Their knees pointed in all directions, none of them were keeping their “knees over toes” with arms stretched out in front as prescribed in a squat jump. Their bodies shook and vibrated. Later during this lesson, the female teacher told me that she expects foolish behavior from these girls.

In this lesson, the teacher prescribed the type of exercises for the students to perform, as well as the number of repetitions and sets. She clearly draws on knowledge from exercise science when telling students how to do their exercises in accordance with established standards: keep your knees over your toes, keep your spine straight, jump up in a straight line, etc. However, three female students of color—smiling and full of life —“wasted” time arranging floor mats instead of beginning the exercises immediately. According to the teacher, the girls were using an inappropriate amount of time to get to work. We interpret their actions as a form of resistance to the teacher's authority. Jane perceived these girls as unwilling to do their exercises, and she told them to “not mess around, and start exercising.” When the girls (finally) started doing squats, their bodies did not adhere to the prescribed exercises, and they seemed to be “bodies out of place” (Azzarito, 2019; Flintoff and Dowling, 2019).

### When Singing and Dancing Are out of Place: A Failure to Understand the “Rules”

As we mention above, we believe that female students of color were unwilling to engage in the exercise activities prescribed by the teachers, and resisted doing so. We observed several situations where female students of color sang, danced, or didn't understand “the rules” when they were supposed to be carrying out strength exercises (they performed them incorrectly on purpose). In one of these lessons, Danish stickball was the activity, and as the teacher had explained at the beginning of the lesson, one of the rules in the game was that if people get caught (somebody throws the ball and hits them), they must perform a penal activity (i.e., ten sit-ups). Performing the penal activities allows one to enter the game anew. The pedagogic use of this game and rules related to penal activities, were applied to keep the students at a high activity level that promotes the training of endurance and strength. At this school the teachers always played loud music during PE lessons. During the Danish stickball activity, the first author registered that all the girls were wearing hijabs, and some of them were also garbed in long dresses. They sang and danced while they participated in the game, even though this was not a prescribed part of the activity. They reached out their hands and shook their upper bodies. But they also performed the “penal activity” when they got caught. During the Danish stickball some of the girls remained seated on the floor, talking, while the game was still going on. Jane saw this. Seeing pupils sitting affected her and she shouted at them, waving her arms to signal to the girls that they should get up. “What is going on with the people sitting there?” she asked. Nothing happened. She walked over to the girls, said something to them, and the girls immediately started to do sit-ups. When the first author asked Jane what she had said to girls, she responded that she had asked them: “Why are you still sitting?” Their response had been that they “got tagged”. Jane said that the pupils had failed to understand the rules.

The lively girls had a lot of energy and appeared to enjoy expressing themselves bodily by dancing and singing along to the music. At the same time, we interpret the girls' foolish behavior, their singing and dancing, and their “failure to understand the rules” as an expression of their unwillingness to take part in training activities they consider pointless and boring.

### Dance Instructions and Preferences

It was interesting to observe the students dancing, in part because of the confrontations that occurred between “legitimate” dance (from a selected Western repertoire of steps and movements) and female students of color's preferred ways of dancing. In the following situation, dance was the teaching content in the lesson, consisting of shuffle dance, jig circle, line dance and hip hop. Thomas started to show the steps in a shuffle dance. Four of the female students of color started a conversation about not wanting to take part in the dancing lesson. We observed that they discussed taking the lowest grade instead, saying that they could just position themselves on the sideline and watch. Jane also noticed that the females were sitting on the sideline, and she came over to them. They told her that they did not want to dance. Jane commented that it is important to participate, and she assured them that there was no couple dancing. She argued that participation was a big part of the assessment and the grading, and let her words hang in the air “It is up to you.” The four females had a short discussion together, and shortly after they joined in the dancing lesson. Nevertheless, their bodies displayed resistance through their lack of energy and enthusiasm.

In this situation the four female students collectively refused to participate in the lesson and dance. Jane responded to the girls' refusal with the comment “there is no couple dancing,” presumably interpreting their unwillingness to mean that the girls didn't want to participate because they were concerned about body contact with other students. We did not observe any other communication between the students and Jane before she concluded by saying “it is up to you.” However, she made the consequences clear, saying that if they did not take part in the lesson, they would get lower grades in PE. In other words, Jane made the students an offer they couldn't refuse. These girls have no real option except to participate in the dances if they care about their assessment and grading in PE. In this lesson, all the dances originate in Western countries. Dances from non-European cultures are not a prominent part of the PE content, thus marginalizing students with interest or experience in those “other” types of movement. In this situation, the four female students of color resisted taking part in the lesson, until they were threatened with low grades, and finally pressured to participate. However, they still showed resistance by displaying disengagement during the lesson.

One other situation demonstrates how female students of color preferred to dance. Often when “obligatory” exercises were completed, students could choose between different activities during the rest of the lesson. In such “free time situations” many of the female students of color would often “hide” in an adjoining mirror-room, and dance to popular Indian music. The first author was fascinated by seeing the four female students dancing synchronically to Indian music with a strong rhythm. One of them stood in front of the other. In general, the dance moved progressed by lifting one foot slightly high, and one step is taken backwards making the feet aligned. The move back and forth and repeat the foot lifting again. At the same time various arm movements were performed. It's all rhythmical and retains a high tempo throughout the dance. They kept it going for 30–45 seconds, then halting, conferring, instructing each other, and performed new elements of the dance.

The energy was high, and a shared flow filled the space with movements the girls *enjoyed* doing, with dedication and a high level of competence. They created their own space and time full of joy and energy. While the pupils performed “their own activity,” the teachers sat downstairs in the sport hall, observing the pupils that played various ballgames. Occasionally they made a trip upstairs to the fitness room and the mirror room to “check” that the students were busy. We noted that the teachers only gave recognition and positive feedback to the pupils that trained hard during the lessons, and to pupils who displayed developed sport skills in traditional ballgames. The dancing female students in the mirror-room received little, if any, recognition for their dancing competence as far as we could see.

## Discussion

In our findings, the female students of color expressed themselves actively, but often at odds with the expected norms of intensity, activity and performing exercise from the health and sport discourses. Even though they were clearly full of vitality and expressiveness, these students came to represent bodies out of place in the established (white) PE culture. The prescribed exercises support an objective and outward-oriented concept of measurable movement and underestimate the significance of other movement experiences that are not part of the formal class curriculum. The PE teachers give no indication that they recognize that pupils can feel alienated and disconnected from the own bodily learning, and that communication about personal experience transcends conversations about the effect of muscular training or other, similarly mechanistic topics. The teachers' instructions, however, draw on healthism discourses influenced by knowledge derived from exercise physiology and biomechanics, which produce a normative ideal of the body and movement in PE teaching practice. Such practices grant “universal” status to what is a cultural practice for keeping a healthy, physically fit body. This body image is constructed and displayed as the result of a disciplined, conscientious, and effortful adherence to the body workout regime. Bodies that conform to this standard are presented as high-status in PE teaching practice. According to Azzarito et al. (2017), the bodies that are perceived to successfully achieve physical fitness are primarily white bodies. Similarly, Evans (2004) points out that fitness and health practices as such, will afford middle class children advantages (Evans makes a class-based argument, but the advantages he describes are based on race as well as class), that may not be enjoyed in, for example, Muslim homes where a body-centric focus may run counter to religious beliefs and be considered neither a priority nor a proper concern (Evans, 2004; Benn and Phister, 2013). Thus, according to Azzarito (2019), pedagogical practices which focus heavily on instructions for achieving physical fitness will end up defining the bodies of many young, ethnic minorities as “deficient” when measured against normative ideals based on white culture and physiology. Consequently, many young people of ethnic background may struggle to find a positive self-identity, to see and construct their bodies in a positive light in a society and culture that tells them otherwise (Azzarito, 2019).

However, even though our findings shed light on fitness practices that seem to position female students of color as “bodies out of sight” (Azzarito, 2019), bodies that fail to conform to normative ideals of a healthy, physically fit body, we perceived them as vital, playful, and creative. They in no way appear as passive, “invisible” and powerless victims of a discriminatory pedagogical practice (Hill and Azzarito, 2012; Thorjussen and Sisjord, 2018). While many of the female students of color's movements appear clumsy and awkward when engaged in Western exercises such as burpees, squats, and other prescribed physical fitness exercises, these same girls appear rhythmic, confident, and “in synch” with their bodies and movement capability when they are dancing on their own terms. Luckily, these females are given some “space” and opportunities for freedom of movement when the mandatory strenuous exercises are completed. While many of the boys are playing different ball games or doing strength exercises, many of the female students of color were dancing in the mirror-room. However, these students did not always wait until after the required exercises were completed. They sang, danced, and performed exercises in a playful way during the mandatory physical fitness exercises. Sometimes the teachers corrected them, or they were told that they should try to make more of an effort, but often they were “overlooked” by the teachers. Drawing on Ahmed's description of “willful girls,” we interpret these female students' actions as resistance toward what they seem to perceive as “boring” exercises not relevant to their lives. This resistance is apparent when they verbally express that they do not want to take part in (some aspect of) the lesson content, but most of the time it is a “tacit” resistance they show by singing, dancing, or generally showing low levels of engagement during the prescribed exercises. The female students of color's expressions, vitality, and ways of moving contrasted with the values presented in the “Norwegian” style of moving in PE teaching. In the “Norwegian” PE movement culture, enacted by the teachers, a template for biomechanical, medical knowledge was presented and privileged, while students' own experiences of moving, their personal expressions, and their vitality were absent from the lesson plan entirely.

Even though we argue that the physical fitness practices which dominate teaching content in PE classes privilege white students (Azzarito, 2019; Barker, 2019), we realize that many within the category “white” also struggle to adhere to normative ideals of the physically fit and healthy body. Social-economic status as well as race is highly relevant as a determining factor in this issue. Similarly, there is great diversity within the category “female students of color” (Dagkas and Hunter, 2015). Within this group there are differences in religion (Islam, Christianity, Hinduism) and the “strictness” with which one practices religion in everyday life. Some Muslims, for example, wear a hijab in PE lessons, which can pose its own set of challenges when taking part in co-ed PE classes. Furthermore, there are huge cultural and social-economic differences between students from different countries in Africa, Asia, and Europe. Nevertheless, due to the healthism discourse that “glosses over cultural differences and, moreover, denies the roles of culture and embodiment” (Azzarito, 2019, 2), we suggest that there is enough commonality across the category “female students of color” to make it relevant and useful in an attempt to shed light on a group of people that are marginalized and exposed to structural discrimination in PE teaching lessons (Hill and Azzarito, 2012; Benn and Phister, 2013; Thorjussen and Sisjord, 2018). Most of these students are not viewed as valued or able bodies within the PE context by either their peers or their teachers. Instead, we find that these students are constructed and positioned as not adhering and conforming to physical fitness exercises, both of which are a central part of the PE teaching content in Norway (Moen et al., 2018), and other Western countries (Kirk, 2010). PE teaching practices that emphasize strenuous exercise as a means of fulfilling what is considered a “physically fit” ideal instead of acknowledging students' bodies and subjectivities functions as a cultural colonization of female students of color's bodies via the enforcement of white male standards (Azzarito, 2019). Such practices are counterproductive in terms of the Norwegian school system's overall goals of inclusivity, creativity, and enjoyment.

We acknowledge that our prejudices and methodology have influenced our production and analysis of the empirical material (Miller and Fox, 2003). In this regard, our findings show one possible interpretation of the “encounter” between institutionalized pedagogical practices and female students of color in Norwegian PE. However, based on our research question and our empirical material, we have selected situations which we believe “unmask” (Jørgensen and Phillips, 2002, 185) some widespread assumptions about how bodies and movements should be evaluated in PE teaching practice. Moreover, we think that these situations—showing how female students of color move and express themselves in PE lessons—add something “new” to the research literature.

## Conclusion

In this article, we show how PE teaching practices in a multicultural school in Norway emphasize the development of the students' physical fitness. The content in PE lessons puts an enormous weight on physical fitness training, and the teachers' communication to the students shows that they value exercises and activities that produce a level of intensive physical activity. It exposes a hegemonic belief that intensive physical exercise = becoming physically fit = good health. This conception of health appears as a normalized universal truth in PE and is based on a “one size fits all” way of thinking. In this context, bodies that adhere to the desired standard of physical fitness have more value than those that do not. Like many other scholars, we argue that such hegemonic “truths” about the body, health and movement make teachers “blind” to how PE teaching practices privilege ethnically white male students (Azzarito, 2019). In the eyes of the teachers, the female students of color's movements and bodies are seen as “deficient” and “out of place.” Our findings show how these female students are faced with discriminatory practices in PE, but they also show how these students resist the hegemony of the culturally dominant PE teaching practices. In contrast to the teachers, we see these female students' bodies as rhythmic and confident with themselves. We see students who express vitality, spontaneity, creativity, and joy. Such expressions are perfectly in accordance with the curriculum document, emphasizing that PE should encourage spontaneous activity and stimulate experimental and creative development. We believe that if these movements and expressions that are overlooked and marginalized by the PE teachers were treated with greater respect and incorporated into the class content, they could contribute great value to physical education in Norway. Moreover, as many Western countries are becoming more multicultural societies with an increased awareness of structural discrimination and racism, we must learn to pivot away from stereotypical movement practices that define and categorize students of color's movements and bodies as deficient or “out of place.”

## Data Availability Statement

The raw data supporting the conclusions of this article will be made available by the authors, without undue reservation.

## Ethics Statement

The studies involving human participants were reviewed and approved by Norwegian Center for Research Data. Written informed consent to participate in this study was provided by the participants' legal guardian/next of kin.

## Author Contributions

All authors listed have made a substantial, direct and intellectual contribution to the work, and approved it for publication.

## Conflict of Interest

The authors declare that the research was conducted in the absence of any commercial or financial relationships that could be construed as a potential conflict of interest.

## Publisher's Note

All claims expressed in this article are solely those of the authors and do not necessarily represent those of their affiliated organizations, or those of the publisher, the editors and the reviewers. Any product that may be evaluated in this article, or claim that may be made by its manufacturer, is not guaranteed or endorsed by the publisher.

## Footnotes

^1^In November 2020, students from Outdoor Studies accused their university of perpetuating structural racism in its curriculum. The students demanded a decolonization of the curriculum by increasing conscious reflection and examination of the content, as well as inclusion of more non-western perspectives (Trædal, 2020). Furthermore, a new report addresses concerns about structural racism in Norway's National Sport Organization (Kristiansen and Sonne, 2021).

^2^Until 2020, due to the negative connotations of the word “race” since World War II, researchers in the fields of anthropology and sociology have instead preferred to study ethnic groups and minority identities within the modern nation state. Rather than treat race as a biological reality, they address *racialization*, the categorization of people based on characteristics that are assumed to be innate in racism (Gullestad, 2004; Svendsen, 2014).

^3^We struggled to name this category. We identified different conceptions in the literature: ethnic minority, non-white, multiethnic, black and minority. However, some consider the phrase “ethnic minority” to be hurtful or offensive. We chose to employ the same denomination that Ruth Frankenberg and Sarah Ahmed use in their works. However, due to our chosen critical whiteness perspective, we remain ambivalent to naming/defining a category in such a way that it reproduces or perpetuates the idea of “the other”.

^4^We consider the word ≪Western≫ to be too diffuse to be useful. There is a huge diversity in “western” thought, even when one limits the discussion to continental philosophy, which includes the work of a wide array of thinkers, from Hanna Arendt to Sarah Ahmed.

^5^A new curriculum was implemented in Norway autumn 2020. This study was conducted during the former curriculum. The presentation of the curriculum is based on the former curriculum.

^6^This article draws on empirical material from first author's Ph.D. project, in which he investigated how PE is constituted in teaching practice at four upper-secondary school located in Oslo, Norway. He conducted ethnographically inspired fieldwork combined with a discourse perspective (Miller and Fox, 2003). This means that he paid attention to what was said and done in PE teaching practice, and these “sayings” and “doings” were seen as “expressions of culturally standardized discourses that are associated with particular social settings” (Miller and Fox, 2003, 44).
